# Exploring the mechanism of trait depression and cognitive impairment on the formation of among individuals with methamphetamine use disorder under varying degrees of social support

**DOI:** 10.3389/fpubh.2025.1435511

**Published:** 2025-02-06

**Authors:** Wei Wang, Guangsheng Sun, Chen Li, Cunxi Qiu, Junyi Fan, Yuhan Jin

**Affiliations:** ^1^Department of Drug Prohibition and Public Security, Criminal Investigation Police University of China, Shenyang, China; ^2^Key Laboratory of Drug Control Technology in Liaoning Province, Shenyang, China

**Keywords:** methamphetamine use disorder, suicidal ideation, mechanism, trait depression, cognitive impairment (CI), social support

## Abstract

**Background:**

Methamphetamine stands as one of the most widely abused drugs globally. Methamphetamine Use Disorder not only impairs the physical and mental wellbeing of addicts but also elevates their risk of suicide. Despite the high suicide rate among individuals with methamphetamine use disorder, research on their clinical characteristics and suicide risk factors remains scarce. Therefore, it is imperative to investigate the risk factors associated with suicidal ideation in individuals with methamphetamine use disorder.

**Methods:**

Employing Respondent Driven Sampling (RDS), a total of 11,825 individuals with methamphetamine use disorder were selected from April to May 2023 in Guangdong, China. The individuals with methamphetamine use disorder were assessed using the Beck Scale for Suicide Ideation (BSSI), and the detection rate of suicidal ideation among these patients was 23.92%. The Bayesian Mindsponge Framework (BMF) analysis was utilized to examine the risk factors for suicidal ideation among these individuals.

**Results:**

The result revealed that trait depression and cognitive impairment are positively correlated with suicidal ideation in people, whereas social support has a moderating effect on the relationship between trait depression and cognitive impairment with suicidal ideation.

**Conclusion:**

Suicidal ideation in individuals with methamphetamine use disorder is influenced by a multitude of factors, including family, society, and stress. Consequently, comprehensive intervention measures are essential to address this issue.

## 1 Introduction

Methamphetamine ranks among the most widely abused drugs globally, posing a significant threat to human health and safety (1–3). The 2022 United Nations World Drug Report revealed that 34 million people aged 15–64 worldwide used amphetamine-type stimulants in 2020 (4). The addiction issues stemming from methamphetamine abuse, particularly methamphetamine use disorder, have emerged as a pressing public health concern worldwide. Methamphetamine use disorder is characterized by a spectrum of cognitive, behavioral, and physiological symptoms resulting from methamphetamine use. Despite recognizing the potential harms, people who use methamphetamine persist in their habit, indicating a compulsive tendency where susceptible individuals continue to use methamphetamine, disregarding the adverse consequences. According to the Diagnostic and Statistical Manual of Mental Disorders, 5th Edition (DSM-5) (5), stimulant use disorder leads to clinically significant impairment or distress, manifested by increased intake, tolerance, difficulty in controlling intake, severe withdrawal symptoms and negative effects during attempts to quit, including symptoms such as intense drug seeking and craving.

Methamphetamine use disorder not only impairs the physical and mental health of addicts (6) and facilitates the spread of AIDS and other infectious diseases, but it also significantly elevates the risk of suicide (7). Research has demonstrated that people who use methamphetamine are 4.4 times more likely to attempt suicide compared to those who do not use methamphetamine (8). Moreover, individuals with methamphetamine use disorder face a standardized suicide mortality rate that is as high as 17 times that of the general population (9). A comprehensive meta-analysis of mortality rates among people who use methamphetamine revealed a 12-fold increase in the risk of suicide compared to the general population (10). In a recent large-scale cohort study conducted in Taiwan, China by Lee et al. (11), it was found that the standardized mortality rate for suicide among individuals with methamphetamine use disorder was alarmingly high, reaching 16 times that of the general population. Notably, methamphetamine is frequently detected in the blood or urine samples of suicide victims, further implicating it as a proximate risk factor for suicide (12).

There is no consensus on the definition of suicidal ideation. Some scholars contend that it merely encompasses an individual's inclination to end their own life. In contrast, others argue that it signifies a strong desire and motivation to terminate one's existence, along with the contemplation of suicide methods. Additionally, some experts believe that the formulation of suicide plans should also fall under the umbrella of suicidal ideation (13). Suicidal ideation serves as a risk factor for suicide, potentially exerting long-lasting effects (14), and thus, it can be characterized by both state and trait attributes. Two prominent theories can elucidate the emergence of suicidal ideation. The first is the quality stress model, proposed by Schotte and Clum (15), which posits that stress alone is not sufficient to trigger suicidal ideation. Even under identical stress conditions, many individuals do not develop suicidal thoughts or behaviors. The risk of suicidal ideation and behavior escalates only when stressed individuals already possess certain susceptibility traits. Another iteration of the quality stress model, introduced by Mann et al. (16), clarifies the concepts of stress and quality. Stress pertains to mental and psychological disturbances, whereas quality represents trait-based psychological structures, such as an impulsive personality. The second theory is the Integrated Motivation-Volitional Model (IMV) (17), which categorizes the occurrence of suicidal behavior into three distinct stages: pre-motivational, motivational, and volitional. The pre-motivational stage encompasses factors like personal “qualities,” external environmental influences, and life events. The motivational stage is marked by feelings of frustration, shame, and entrapment, which progress to the emergence of suicidal ideation. The volitional stage, building upon suicidal thoughts, involves acquiring methods or means for suicide, exposure to suicide-related information, or impulsive actions, ultimately leading to the act of suicide.

Both theories offer valuable frameworks for researchers to examine the development of suicidal thoughts and behaviors systematically. However, to gain a deeper understanding of the root causes of suicidal ideation, it is essential to consider not only the interplay of internal risk factors but also potential underlying mechanisms. The human mind is a complex and multifaceted system, so its psychological processes are best explained and interpreted through dynamic models rather than traditional static theories. Furthermore, when studying the specific population who use drugs, investigating the distribution and determinants of suicidal ideation necessitates a focus on exploring the psychological mechanisms behind suicidal ideation and how specific environmental factors affect the thinking of this population.

As a unique comprehensive analysis tool, the Bayesian Mindsponge Framework (BMF) integrates the essence of the Mindsponge mechanism and Bayesian theory. The mindsponge theory explains how internal information processing and external information exchanges influence human psychology and behavior. This framework stems from the metaphysical perspective that physical reality is composed of information, expressed hierarchically as Information—Laws of Physics—Matter, in contrast to the traditional view of Mathematics—Physics—Information (18). Mindset is a collection of core values (or perceived important information) that are the benchmark for the information-processing mechanism (19). In this case, the negative feelings induced by drug seeking and craving can be seen as contributors to internalizing suicidal ideation (or suicide-related information) into the mindset by linking more values to the act of suicide (e.g., stop the suffering). The appearance of suicide-related information in the mindset does not necessarily lead to suicide behavior. However, it increases the risk of suicide. When a certain threshold is passed (i.e., suicide-related information is considered so important and valuable that it surpasses the desire to live its pertinent benefits), the act will happen. The mechanism is a non-stop, continual absorption and ejection process of information that aims to maximize the person's perceived benefits and minimize the perceived costs. The mindsponge theory has recently been updated by incorporating quantum mechanics knowledge and Shannon's information theory, further improving its suitability to explain psychological issues from cognitive perspectives and flexibility in connecting existing theories and frameworks (20).

Building on this foundation, this study intends to utilize the Mindsponge mechanism to delve into the cognitive and environmental interplay that underlies suicidal ideation in this specific population who use methamphetamine. The ultimate goal is to provide a scientific basis for developing strategies and interventions aimed at preventing methamphetamine use disorder and suicidal behaviors, thereby promoting overall health and wellbeing.

## 2 Methods

### 2.1 Study population

From April to May 2023, a multi-stage stratified random sampling approach was employed, stratifying by region and economic level. One city—Guangzhou, Shantou, Qingyuan, or Maoming—was randomly chosen from the eastern, western, northern, and Pearl River Delta regions of Guangdong Province, respectively. Within each selected city, three districts (or counties) were randomly picked, followed by the random selection of three townships (or streets) from each district. Subsequently, 165 people who use methamphetamine were sampled from each township using Respondent Driven sampling. RDS, an enhancement of the long-chain recommendation method, relies on peer responses. Initially, methamphetamine use disorder, identified through the local people who use drugs dynamic control system, were recruited as seeds for the survey. Trained investigators conducted face-to-face questionnaire surveys with these seeds. Seeds received recruitment cards and commemorative gifts (junior incentives) upon completion. They were then tasked with recruiting peers residing in the same township, providing them with peer recruitment cards. Each seed could recommend up to five peers. Peers, bearing recruitment cards, visited designated survey locations where investigators assessed their eligibility for the study. Eligible participants provided informed consent and completed the questionnaire. They, too, received recruitment cards and junior incentives. For each successful recruitment of a peer from the same township, recruiters earned additional commemorative gifts (secondary incentives). This cycle continued through multiple recruitment rounds, with the sample's characteristic variable composition stabilizing over time. Recruitment ceased once a representative and seed-independent sample was obtained.

Upon admission, all participants underwent urine toxicology screening and self-reporting to confirm people who use methamphetamine. A diagnosis of methamphetamine use disorder was established through semi-structured clinical interviews conducted by a professional psychiatrist with at least 5 years of clinical experience, utilizing the DSM-V, Axis I, Patient Version, and the Chinese version of the SCID-I/P. To be included in the study, participants had to meet the following criteria: (1) They met the diagnostic criteria for methamphetamine use disorder in both the International Classification of Diseases, Tenth Version (ICD-10) and the Diagnostic and Statistical Manual of Mental Disorders, Fifth Edition (DSM-V); (2) They were residents of Guangdong with a local household registration or had resided in Guangdong for more than 6 months; (3) They had been abstinent from drug use for <1 year by May 30, 2023, whether they were still socially active, had been forced into drug rehabilitation centers, or had voluntarily detoxified; (4) They were aged between 18 and 60 years, with an average age of 28 years; (5) They had no history of diabetes or specific nervous system diseases, including head trauma and epilepsy. Exclusion criteria included patients unwilling to continue participating.

The Beck Scale for Suicide Ideation (BSSI) (21) was utilized to assess suicidal ideation among individuals with methamphetamine use disorder. The final investigation encompassed 11,825 individuals with methamphetamine use disorder, based on the total weighted score of the BSSI, 2,828 suicidal ideation among these patients were detected. All participating patients received approval from the Ethics Committee of the School of Narcotics Control and Public Security, Criminal Investigation Police University of China (2023-12) and signed informed consent forms.

### 2.2 Study tools

(1) The Beck Scale for Suicide Ideation (BSSI) (21) was employed to assess the suicidal ideation and tendencies of methamphetamine use disorder. Developed by Beck et al. in 1979, the BSSI is a highly regarded tool for evaluating the intensity of specific attitudes, behaviors, and plans related to suicide. Originally designed as a self-assessment tool for psychiatric patients during interviews, it has since been widely adopted for use in non-psychiatric populations and can also function as a self-screening tool for suicide risk (22). The BSSI comprises 19 items, divided into two dimensions: suicide ideation (SI, items 1–5) and suicide tendency (ST, items 6–19). Each item is rated on a 3-point Likert scale based on the intensity of suicidal thoughts: 0 = no thoughts, 1 = mild thoughts, and 2 = moderate to strong thoughts. The item scores are summed to produce a total score ranging from 0 to 38. The scale covers various aspects of suicide, including the desire to die, active or passive suicide attempts, the duration and frequency of suicidal ideation, perceived control over suicide attempts, and the extent of preparations made for suicide. Most participants complete only the first five items, known as the suicide ideation subscale. If both items 4 and 5 receive a score of 0, the individual is considered non-suicidal and does not need to complete the suicide tendency dimension. A non-zero score for items 4 or 5 indicates suicidal ideation, prompting further evaluation of the suicide tendency dimension. In the methamphetamine use disorder questionnaire, the BSSI demonstrated strong internal consistency, with an overall Cronbach's α coefficient of 0.93 and subscale Cronbach's α coefficients ranging from 0.84 to 0.90.

(2) A self-designed general information survey questionnaire was utilized to gather data on various demographics and background information, including gender, age, education level, marital status, occupation, whether the participant is an only child, whether they were primarily cared for by their parents during childhood, and family history of mental illness, among other relevant factors.

(3) The Trait Depression Scale (T-DEP) is a subcomponent of the State-Trait Depression Scale (ST-DEP), developed by Charles D. Spielberger in 1995. It assesses an individual's long-standing depressive mood (trait), rather than their transient state over a 1- or 2-week period. Notably, the ST-DEP omits somatization-related items and focuses on cognitive and emotional aspects, which are further categorized into two factors in both the State Depression Scale (S-DEP) and T-DEP: euthymia and dysthymia. Euthymia denotes “the presence of positive emotions,” whereas dysthymia signifies “the presence of negative emotions” (23). Importantly, the Euthymia subscale uses a reverse scoring system, indicating a “lack of positive emotions” or “anhedonia.” The T-DEP consists of 32 items, evenly split between S-DEP and T-DEP. Within the 16 T-DEP items, eight reflect anhedonia, and the remaining eight pertain to mood disorders. In this study, the internal consistency coefficients for the T-DEP subscales of anhedonia and mood disorders were 0.910 (95% CI = 0.892, 0.928) and 0.861 (95% CI = 0.831, 0.892), respectively, demonstrating strong internal consistency.

(4) The Multidimensional Scale of Perceived Social Support (MSPSS) (24) was utilized to gauge the level of social support among individuals with methamphetamine use disorder. Developed by Zim in 1988, the MSPSS has since been translated into multiple languages and widely adopted globally as a standard measure of perceived social support. The scale comprises 12 items, assessing support from three dimensions: family support (FamS: items 3, 4, 8, and 11), friend support (FriS: items 6, 7, 9, and 12), and significant other support (SoS: items 1, 2, 5, and 10). Each item is rated on a 7-point Likert scale based on the level of agreement: 1 = strongly disagree, 2 = moderately disagree, 3 = slightly disagree, 4 = neutral, 5 = slightly agree, 6 = moderately agree, and 7 = strongly agree. Higher total scores indicate stronger perceived social support, with a range of 12–84. Scores of 12–48 suggest low social support, 49–68 indicate moderate support and 69–84 reflect high social support. In this study, the MSPSS demonstrated excellent internal consistency in the methamphetamine use disorder questionnaire (Cronbach's alpha = 0.89).

(5) The Montreal Cognitive Assessment Scale (MoCA) (25), developed by Nasreddine from Canada, is grounded in clinical experience and incorporates cognitive items and scoring criteria from the Mini-Mental State Examination (MMSE). The MoCA evaluates cognitive function across several domains: visual-spatial and executive abilities (5 points), naming (3 points), attention (2 points), reading and writing skills (1 point), computation (3 points), language ability (3 points), abstract thinking (2 points), delayed recall (5 points), and orientation (6 points), with a total score of 30 points. A score of 26 or above is considered normal, while lower scores indicate more severe cognitive impairment.

The respondents themselves completed all survey questionnaires after receiving instructions from uniformly trained investigators on how to fill them out. The completed questionnaires were then collected immediately.

### 2.3 Statistical analysis and variables

In the current study, we utilized individual traits, social support, and cognitive status (with specific variables outlined in Table 1) to develop the Bayesian Mindsponge Framework. The framework is represented as follows:

**Table 1:** Variable's description.

**Variable**	**Meaning**	**Study tools**	**Data type**	**Value**
Suicide	Suicide ideation	The Beck Scale for Suicide Ideation (BSSI)	Binary	1 = Yes 0 = No
Traits	Traits depression	The Eysenck Personality Questionnaire Short Scale (EPQ-RS)	Binary	1 = Yes 0 = No
Support_L	Low social support	The self-designed methamphetamine addiction degrees diagnostic scale	Binary	1 = Yes 0 = No
Support_M	Moderate social support	The self-designed methamphetamine addiction degrees diagnostic scale	Binary	1 = Yes 0 = No
Support_H	High social support	The self-designed methamphetamine addiction degrees diagnostic scale	Binary	1 = Yes 0 = No
Cognitive	Cognitive impairment	The Montreal Cognitive Assessment Scale (MoCA)	Binary	1 = Yes 0 = No

Suicide~α+ Traits + Cognitive + Support_L ^*^ Traits + Support_M ^*^ Traits + Support_H ^*^ Traits + Support_L ^*^ Cognitive + Support_M ^*^ Cognitive + Support_H ^*^ Cognitive.

This model is designed to investigate how traits and cognition impact suicidal ideation among individuals with methamphetamine use disorder, considering varying levels of social support (low, medium, and high).

Statistical analysis was performed using R software (version 4.4.1), specifically leveraging the bayesvl package (26–28). We employed the Pareto smoothed importance-sampling leave-one-out cross-validation (PSIS-LOO) method to assess the Framework's fit to the data. To diagnose the Markov Chain Monte Carlo (MCMC) simulation, we utilized the effective sample size (n_eff) and the potential scale reduction factor (Rhat). Additionally, we analyzed convergence through trace plots, Gelman-Rubin diagnostics, and autocorrelation plots.

## 3 Result

### 3.1 The situation of suicide ideation score of individuals with methamphetamine use disorder

In this study, we assessed suicidal ideation and its various dimensions using the Beck Scale for Suicide Ideation (BSSI) in 11,825 individuals with methamphetamine use disorders. The BSSI scores ranged from 0 to 26, with an average score of 2.59 ± 4.82 and a median of 0. When ranking the two dimensions of suicidal ideation by average score, suicidal tendency (1.49 ± 3.12) was higher than suicidal ideation (1.11 ± 1.94). We determined whether individuals with methamphetamine use disorder had suicidal ideation based on whether the sum of scores in items 4 and 5 of BSSI was 0. Ultimately, 2,828 individuals with methamphetamine use disorder with suicidal ideation were detected, with a detection rate of 23.92%.

### 3.2 The model's goodness-of-fit

**Figure 1:**
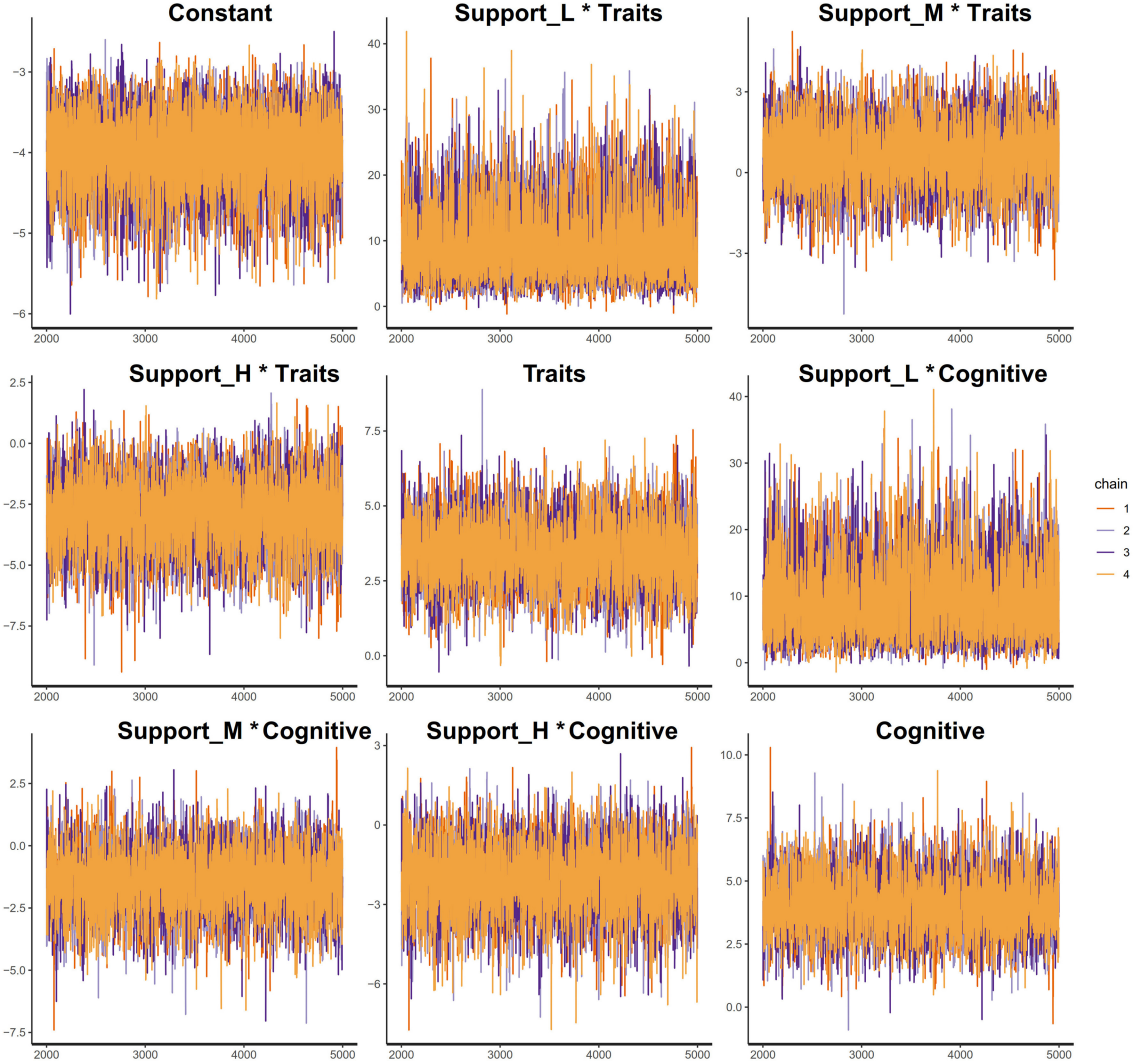
PSIS diagnostic plot.

The model's fit was evaluated using Pareto smoothed importance-sampling leave-one-out cross-validation (PSIS-LOO). The results indicated that all *K*-values for the model were below 0.5, suggesting a strong fit for the Framework (see Figure 1).

### 3.3 The convergence of the MCMC simulation

The MCMC simulation was diagnosed using the effective sample size (n_eff) and the potential scale reduction factor (Rhat), as presented in Table 2. With a n_eff value exceeding 1,000 and a Rhat value of 1, it is evident that the Markov chains have converged.

**Table 2:** Simulated posteriors.

**Parameters**	**Mean**	**SD**	**n_eff**	**Rhat**
Constant	−4.01	0.48	7,699	1
Traits	3.44	1.04	4,667	1
Support_L ^*^ Traits	10.00	5.69	4,726	1
Support_M ^*^ Traits	0.63	1.17	5,228	1
Support_H ^*^ Traits	−2.93	1.40	5,337	1
Cognitive	4.05	1.13	4,601	1
Support_L ^*^ Cognitive	9.52	5.62	5,156	1
Support_M ^*^ Cognitive	−1.47	1.24	5,026	1
Support_H ^*^ Cognitive	−2.70	1.27	5,217	1

### 3.4 Model visual diagnostics

**Figure 2:**
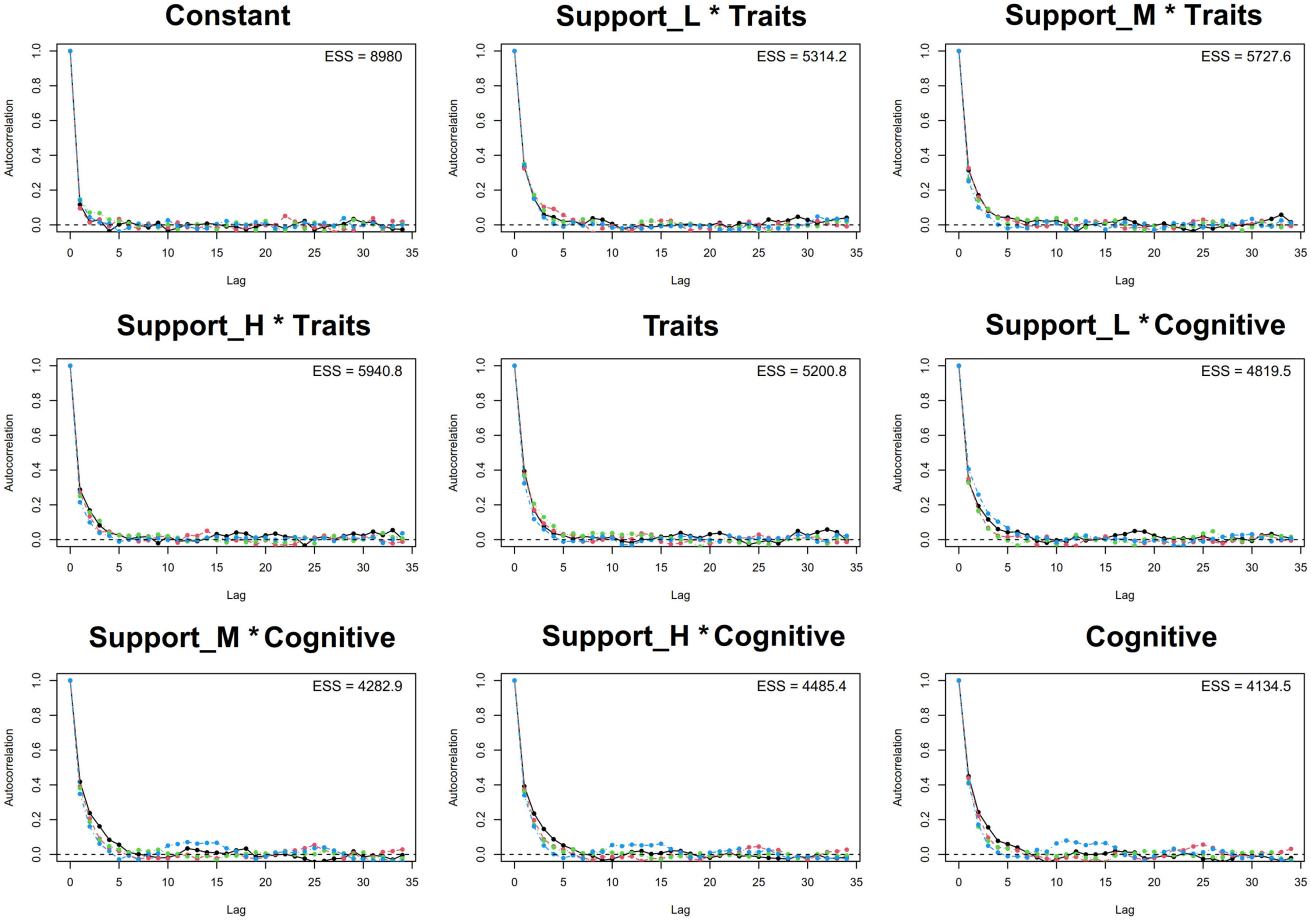
Trace plot and Gelman plot.

The convergence of the MCMC model was further evaluated through the trace plot and autocorrelation plot, as shown in Figure 2. The plots reveal that the parameter trajectories fluctuate around a mean without exhibiting any trends or autocorrelation, indicating that the MCMC fitting results are indeed convergent. This confirms the robustness and reliability of the model's fitting and predictions.

### 3.5 Result of visual presentation

**Figure 3:**
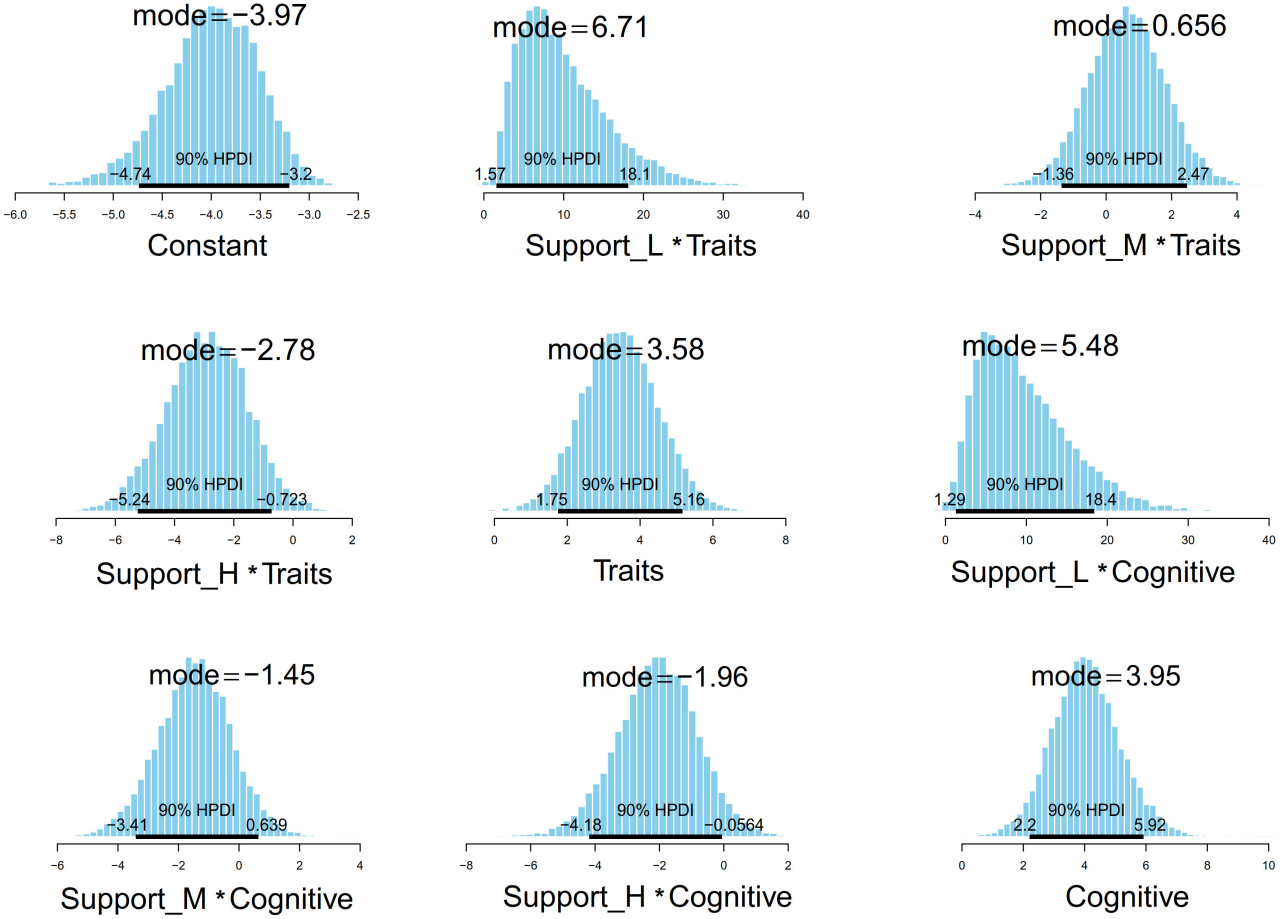
Parameters' posterior distributions with HPDI at 90%.

Figure 3 displays the Monte Carlo simulation results for the posterior distribution of parameters, including the estimated values' mean, median, and 95% confidence interval. Specifically, the average per capita consumption values, as simulated by the Monte Carlo method, for Traits, Support_L ^*^ Traits, Support_M ^*^ Traits, Support_H ^*^ Traits, Cognitive, Support_L ^*^ Cognitive, Support_M ^*^ Cognitive, and Support_H ^*^ Cognitive were 3.64 (90% HPDI: 1.73, 5.17), 5.69 (90% HPDI: 1.86, 18.7), 0.39 90% HPDI: 1.35, 2.46), −2.71 (90% HPDI: 5.13, −0.156), 4.05 (90% HPDI: 2.20, 5.91), 5.66 (90% HPDI: 1.22, 18.10), −1.18 (90% HPDI: −3.44, 0.57), and −1.90 (90% HPDI: −4.18, −0.05), respectively.

**Figure 4:**
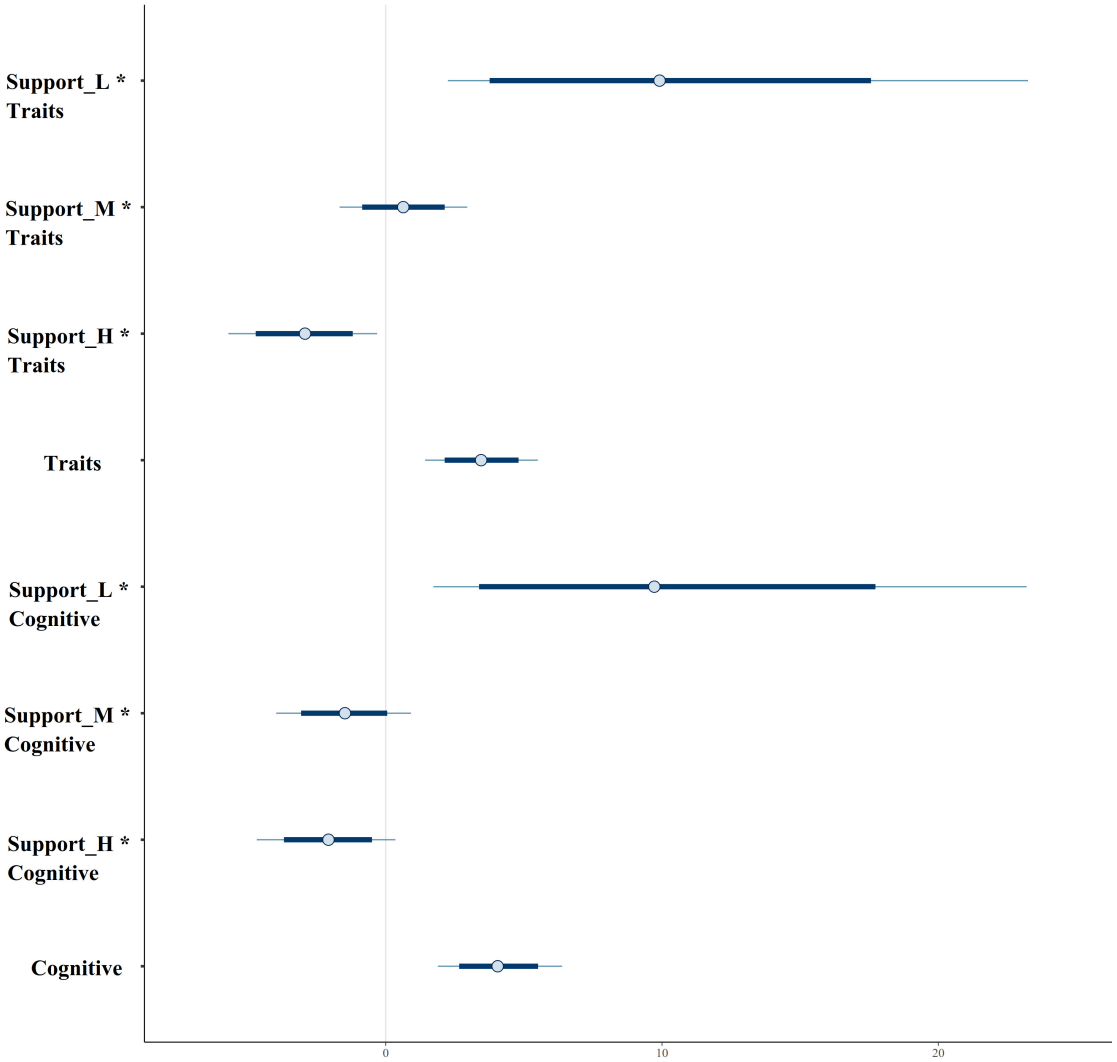
Interval plot of parameters' probability distributions.

Based on the estimation results, we observed that the posterior distributions of Traits and Cognitive are entirely situated on the negative side of the parameter value axis (*x*-axis). This suggests that patients experiencing trait depression or cognitive impairment are more inclined to make decisions that closely align with suicide ideation. Visually, as the level of social support increases, the posterior distribution value also rises (as shown in Figure 4). Notably, the posterior distribution for high social support is consistently lower than that of the other two intercepts. This chart serves as compelling evidence for the reliable impact of social support on the decision to approach suicide.

## 4 Discussion

### 4.1 Insights derived from the model analysis results

In recent years, the issue of methamphetamine abuse has escalated dramatically, posing a significant threat to public health and safety in China. Research has solidly established that prolonged or excessive use of methamphetamine can impair social, cognitive, and executive functions within the human frontal lobe (29), thereby diminishing the brain's control mechanisms and increasing the likelihood of suicidal behavior among people who use methamphetamine.

In this study, involving 11,825 individuals with methamphetamine use disorder, the mean total score for suicidal ideation, as assessed by the Beck Scale for Suicidal Ideation (BSSI), was 2.59 ± 4.82. Specifically, the mean score for the suicidal ideation dimension was 1.49 ± 3.12, and for the suicide plan dimension, it was 1.11 ± 1.94. Furthermore, the detection rate of suicidal ideation among these patients was 23.92%. These findings align with previous reports indicating that substance abuse elevates the risk of suicide (30). Notably, the suicide risk varies among different types of people who use substances (30), with methamphetamine use disorder exhibiting a particularly high risk compared to people who use other substances, a fact that has garnered considerable research attention (7).

In this study, we employed the Bayesian Mindsponge Framework analysis to delve into the application of the mindsponge theory to explain the formation of suicidal ideation among individuals with methamphetamine use disorder through an information-processing lens. Our findings reveal that the preexisting depression traits in the mind among individuals with methamphetamine use disorder facilitate the internalization process as it exhausts an individual's positive emotions, further enhancing the perceived values of suicidal acts and reducing the values of continuing their existence. The cognitive impairment reduces the mind's information-processing effectiveness (e.g., problem-solving and coping skills), exacerbating the negative feelings and conditions induced by drug abuse and depression traits. As a result, when the negative feelings and conditions get worse, the values of living decline, whereas the values of suicide increase as suicide is increasingly considered as a problem-solving alternative (e.g., stop the suffering). Meanwhile, social support can be seen as the flow of positive information that helps neutralize the negative impacts of depression trait by relieving the negative feelings and reducing the perceived cost of continued existence.

Specifically, trait depression was found to be positively associated with suicide ideation in individuals with methamphetamine use disorder, suggesting that a depressive personality trait serves as a risk factor for suicidal ideation. This aligns with conclusions drawn from other studies (31, 32). Such personality traits often manifest as high self-demand, rigor, guilt, low self-esteem, and communication difficulties. Methamphetamine use disorder possessing these characteristics is, on the one hand, more susceptible to feelings of despair when faced with external pressures and environmental challenges, which can precipitate suicidal ideation and behaviors. On the other hand, their lack of social skills and difficulty in maintaining healthy social networks and interpersonal relationships hinder their ability to access crucial social and psychological information needed to cope with adverse life events, thereby increasing their vulnerability to suicidal tendencies.

Cognitive impairment is positively correlated with suicidal ideation among individuals with methamphetamine use disorder. A growing array of evidence from related studies (33) supports the notion that cognitive dysfunction may serve as a predictive neurocognitive biomarker for identifying individuals at high risk of suicide. Methamphetamine use disorder is particularly susceptible to cognitive impairment. A mini-review of cognitive deficits linked to methamphetamine use disorder revealed that most people who use methamphetamine experience moderate cognitive impairment, notably in impulse control, reward processing, and social cognition (34). The correlation between methamphetamine use disorder and cognitive impairment has been well-documented (35). This may stem from a lack of knowledge about addiction-related information and cultural awareness regarding mental health issues among individuals with methamphetamine use disorder. Negative perceptions of addiction and mental health issues can further exacerbate this problem, as people who use methamphetamine may fear social stigma, opposition, and discrimination, leading them to have limited access to formal information and assistance related to these issues compared to the general population (36, 37).

On the other hand, social support exerts a moderating influence on the relationship between trait depression, cognitive impairment, and suicide. Under varying levels of social support, the associations between trait depression and cognitive impairment with suicide ideation can either intensify or diminish. For instance: Under low social support, the impacts of trait depression and cognitive impairment on suicide ideation are amplified. Conversely, under medium or high social support, these impacts are mitigated or reduced. As a positive factor in mitigating suicidal behavior, social support helps alleviate psychological stress and dispel irrational thoughts of escaping reality through suicide among individuals with methamphetamine use disorder.

In summary, collaborative efforts from the government, society, and families are imperative to address the issue of suicidal ideation among individuals with methamphetamine use disorder. Firstly, the government and society should enhance the public's ability and awareness to access health information, diminish social discrimination, and offer policy support for vocational training, psychological counseling, and other essential services tailored to individuals with methamphetamine use disorder. Secondly, families and local communities must provide appropriate mental health counseling and support, embrace individuals with methamphetamine use disorder back into family life, help them reshape their misconceptions about addiction and mental health, elevate their subjective perception levels, organize educational activities, build communication bridges, gain a deeper understanding of their mental health status, alleviate negative emotions, and foster a healthy and positive outlook on life. Lastly, at the individual level, it is crucial to strengthen self-regulation skills, enhance mental health education, and develop interpersonal abilities. By engaging in sports and adopting an optimistic mindset, individuals with methamphetamine use disorder can improve their health motivation, reduce anxiety related to their addiction, and effectively prevent the onset of suicidal ideation.

### 4.2 Limitations of this study

Firstly, this study employs a cross-sectional design, which allows us to establish only correlations between the independent and dependent variables; causal relationships cannot be determined. Future longitudinal studies are necessary to further elucidate these causal links between influencing factors and the outcome variable. Secondly, the data for this study were sampled solely from four cities in Guangdong Province, China, resulting in a relatively narrow research scope. Consequently, the conclusions drawn may be specific to the socio-cultural characteristics of these study sites, and their applicability to other regions remains unverified.

## 5 Conclusion

In this study, we delve into the mechanisms underlying trait depression, cognitive impairment, and social support in the emergence of suicidal ideation among individuals with methamphetamine use disorder, grounded in the mindsponge theory. Our findings reveal that trait depression and cognitive impairment exhibit a positive correlation with suicidal ideation in individuals with methamphetamine use disorder. Meanwhile, social support serves as a moderating factor, influencing the relationship between trait depression, cognitive impairment, and suicidal ideation. Consequently, it is crucial to mobilize efforts from the government, society, and families to tackle the pressing issue of suicidal ideation in the context of individuals with methamphetamine use disorder.

## Data Availability

The original contributions presented in the study are included in the article/supplementary material, further inquiries can be directed to the corresponding author.
